# A perspective on harmonizing diabetes management datasets

**DOI:** 10.1016/j.dib.2025.111399

**Published:** 2025-02-17

**Authors:** Miriam K. Wolff, Sam Royston, Anders L. Fougner, Hans Georg Schaathun, Martin Steinert, Rune Volden

**Affiliations:** a Norwegian University of Science and Technology, Department of ICT and Natural Sciences, Larsgårdsvegen 2, 6009 Ålesund, Norway; b Replica Health, 249 Willoughby Ave 4A, Brooklyn NY, United States; c Norwegian University of Science and Technology, Department of Engineering Cybernetics, O. S. Bragstads Plass 2D, 7034 Trondheim, Norway; d Norwegian University of Science and Technology, Department of Mechanical and Industrial Engineering, Richard Birkelands vei 2B, 7491 Trondheim, Norway

**Keywords:** Time series forecasting, Predictive modelling, Blood glucose prediction, Physiological modelling, Standardization, Sensor data integration, Data harmonization

## Abstract

Diabetes management datasets are often compiled from various sensors and devices, including diabetes technology, activity trackers, and other health-related equipment, resulting in heterogeneous data formats. Despite the abundance of available data, inconsistencies in dataset formats and data-sharing practices limit the ability to build on prior work and compare results across studies. Standardizing data-sharing formats can improve consistency, facilitate dataset consolidation, and reduce the data processing burden for researchers. This article explores the current state of data-sharing practices in diabetes management research and proposes guidelines for harmonizing datasets using a unified time-aligned tabular format. We demonstrate the application of these guidelines on three widely used datasets and highlight key challenges in achieving data harmonization. We call on the broader research community to develop and adopt detailed recommendations for standardized data-sharing practices.

## 1 Introduction and Background

Despite notable advancements in diabetes technologies and therapeutic interventions, achieving optimal glycemic control remains a persistent issue. The availability of high-quality diabetes management datasets is essential for data-driven analysis, as well as the development and evaluation of algorithms. A prominent example is blood glucose prediction algorithms, which serve as a critical component of control systems for artificial pancreas devices. Traditionally, these algorithms have relied on physiological models that simulate insulin dynamics and carbohydrate metabolism. For instance, Saiti et al. conducted a comprehensive review of predictive modeling approaches in diabetes, particularly focusing on glucose regulation via model predictive control. Their findings indicate that physiological modeling remains the most reliable approach [1].

However, there has been increasing interest in leveraging machine learning for blood glucose prediction in recent years. This emerging paradigm holds promise for enhancing diabetes management systems and improving patient outcomes [2]. These developments underscore the critical need for standardized, high-quality datasets to facilitate the design, validation, and implementation of advanced diabetes management technologies.

### 1.1 Existing Datasets

Diabetes management datasets are typically collected through observational studies, where real-world data is gathered in uncontrolled environments. Numerous datasets are available in the literature [3,4], with variation in the number of subjects and study durations, ranging from a handful of participants to several hundred and spanning periods from weeks to years. Additionally, these datasets encompass diverse population groups concerning factors such as gender, socioeconomic status, and blood glucose management.

Existing datasets can encompass a variety of features, with continuous glucose monitoring (CGM) and insulin data being the most critical. CGM provides the predicted values, while insulin is the primary regulator of blood glucose levels. Datasets lacking these key components have limited applicability, as they are essential for adjusting insulin dosing and maintaining glucose control. Dietary information is also critically relevant, significantly influencing blood glucose dynamics [5]. This dietary data may be recorded in several ways, including self-reported carbohydrate estimates, image-based assessments, or general meal descriptions that include total grams consumed [6]. Additionally, features from activity trackers or other sensors, as well as manual patient reports, can vary across datasets. Commonly included additional variables of interest comprise data related to exercise, sleep quality, and stress levels, as these factors are known to impact blood glucose dynamics [7]. Such features are increasingly available through non-invasive wearable sensor technology [8].

### 1.2 Data-formatting practices

Existing diabetes management datasets exhibit significant variability in data formatting, terminology, units, scales, and data storage practices. Variability in data sources introduces more inconsistencies, such as sampling rates and recorded values. For example, some datasets pre-divide data into training and test sets, while others organize data by subject or feature. However, a common characteristic is the separation of features into distinct data structures, either within single or multiple files.

**Fig. 1:**
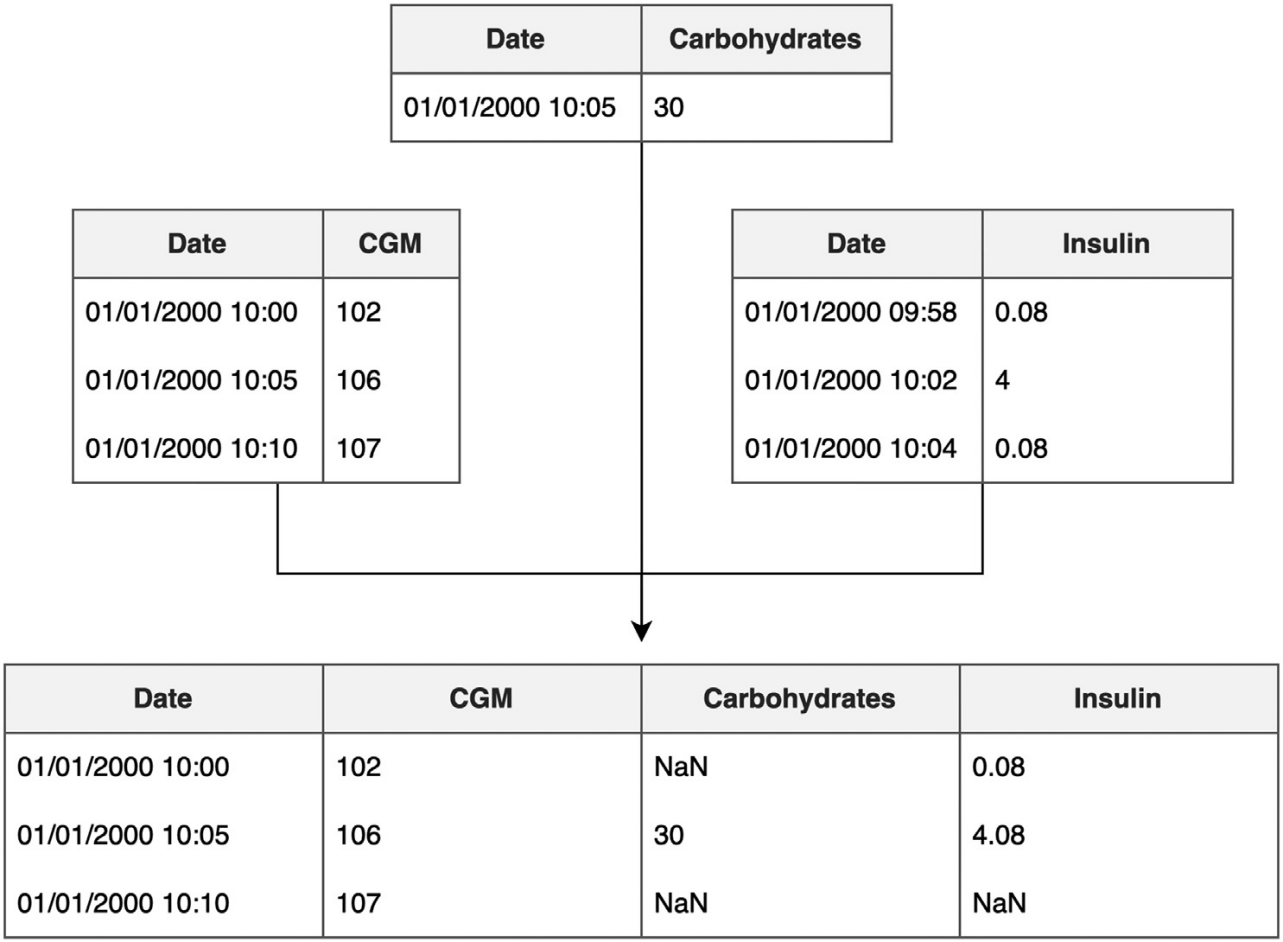
Illustration of resampling features from separate data structures into a unified tabular format, with all features aligned on a consistent time grid.

For blood glucose prediction, aligning features into a cohesive, structured format is essential, rather than storing them as isolated entities. For example, libraries like Scikit-learn and ReplayBG, a digital-twin simulation software for blood glucose, rely on tabular data inputs in their models [9]. Fig. 1 illustrates the process of resampling features, initially stored in separate data structures, onto a uniform grid. For instance, data on carbohydrate intake may be sampled less frequently than CGM and insulin data. As insulin samples may not align precisely with the time points on the uniform grid, we aggregate samples within each time window. This example highlights some of the challenges discussed further in this paper.

Despite the large volume of available data, current data-sharing practices are constrained by inconsistent formats and methodologies. As discussed earlier, there is a significant gap between the typical format of shared datasets and the format required for data analysis or algorithm development. Consequently, researchers often spend considerable time navigating multiple data structures and formats and processing the data before training and evaluating blood glucose prediction algorithms. Standardizing dataset formats would enhance the comparability of studies by reducing discrepancies in data interpretation and processing. Additionally, it would streamline dataset consolidation and integration for algorithm training.

### 1.3 Aim of this study

Current data-sharing practices in diabetes management datasets are constrained by inconsistencies in formats and methods for data organization, which limit comparability and interoperability. In this study, we outline a set of challenges related to aligning diverse features on a consistent time grid in a tabular format. These challenges include managing variations in sampling frequencies, units, terminologies, and other practices specific to time-series data prediction. We propose an initial set of guidelines for data harmonization, demonstrated by processing three common benchmark datasets. These guidelines serve as a foundation and are intended to spark a broader discussion within the research community to refine recommendations, preprocessing techniques, and taxonomies collaboratively. Open-source code accompanies this work to support reproducibility and encourage future contributions.

### 1.4 Recommendations for diabetes management dataset harmonization

#### 1.4.1 Resampling

**Challenge:** Data types are often stored across separate files or structures, yet time series prediction typically requires a tabular format with aligned rows for each feature.

**Suggested solution:** Data providers should consider including a tabular version, as recommended here, where each row represents a fixed time interval. We suggest offering this tabular format alongside the original data to preserve raw data while facilitating model training. Using the CGM sampling frequency as the interval between samples is common, ensuring alignment with the primary data. However, this can be dynamically adjusted in a processing script to suit specific needs, such as matching the temporal resolution required for different models.

#### 1.4.2 Efficient batch processing

**Challenge:** Handling large datasets can be taxing on both processing time and computational resources, especially as file sizes increase. Often, only portions of the data are necessary for specific analyses.

**Suggested solution:** Consider partitioning files when they exceed a predetermined size to facilitate easier access to dataset subsets. The optimal file size for partitioning depends on the storage or processing system, making it important to consult platform-specific best practices.

#### 1.4.3 Modelling of insulin dynamics

**Challenge:** The body's response to different insulin types varies due to differences in their pharmacokinetics, including onset, peak, and duration of action. Although the overall insulin effect may be similar at certain doses, the shape of the absorption curve and the timing of the peak effect differ between insulin types.

**Suggested solution:** Modeling of insulin dynamics supports merging insulin types into a single feature. Additionally, it can enhance blood glucose prediction accuracy [10,11]. For improved blood glucose prediction accuracy, datasets should specify the insulin type each subject uses and include a computed feature for active insulin at each time point, known as “insulin on board” [12]. Given the high computational cost of this calculation, pre-computing it within datasets can help save computational resources.

#### 1.4.4 Variability in scales and units

**Challenge:** The use of scales and units sometimes varies between subjects and datasets. While in a database or similar data structure, you can add units in a separate column, in the tabular format used for algorithms, the unit must remain consistent throughout the dataset.

**Suggested solution:** It is important that dataset descriptions clearly specify the unit used and maintain consistency across the entire dataset. Be aware that certain units or scales may be ambiguous. For example, if “meal in grams” refers to the total grams of food, it could be mistaken for grams of carbohydrates, which is commonly used in this context. Generally, strive for consistency with units used in existing datasets and convert features to the standard unit or scale when possible. For instance, for meals, datasets should at least provide an estimated amount of carbohydrates, as this measure strongly correlates with changes in blood glucose. This estimate might come directly from the subject or be derived from sources such as a photograph of the meal.

#### 1.4.5 Train-test split

**Challenge:** When datasets are not pre-split into training and test sets, researchers may use varying methods for this, complicating study comparisons. They might also overlook best practices for train-test splits in time series data, potentially causing data leakage between splits.

**Suggested solution:** Datasets should be split into training and test sets before being provided to researchers. Best practices for time series splits do not recommend shuffling the data, as this disrupts the time dimension; test data should always follow training data chronologically. Additionally, the common usage of time-lagged features makes it essential to perform the train-test split before creating these features to prevent information leakage from the training set into the test set. Finally, the split should be done per subject, not across subjects, since blood glucose models are often individualized. Without separate training and test data for each subject, it would be impossible to train and test personalized models without relying on online learning methods.

#### 1.4.6 Terminology

**Challenge:** Features may sometimes have unclear or ambiguous names, and identical features can also be labeled differently across datasets.

**Suggested solution:** Datasets should strive to use terminology that aligns with established literature. Feature names should be descriptive, and abbreviations that are not intuitively clear or could be mistaken for other terms should be avoided. For example, “heart rate” is preferable to “hr” or “pulse,” as “hr” could represent something unrelated, and “heart rate” more clearly conveys the measure of heartbeat frequency than “pulse.” However, commonly recognized abbreviations, like CGM, are acceptable when they are widely understood within the domain. Table 2 lists our recommended feature names.

#### 1.4.7 Irregular sampling rates and missing data

**Challenge:** Handling features with variable and irregular sampling rates presents several challenges. Firstly, multiple samples within each time window may need to be aggregated, or low-frequency samples imputed to ensure continuity. Secondly, careful management of resampling is crucial to prevent data leakage. Additionally, for features recorded only on specific events, it can be ambiguous whether missing values indicate a lack of data or simply the absence of any events.

**Suggested solution:** If there are several samples of a feature within the time window, data aggregation is suggested. Data aggregation technique should be chosen based on the data type. The **sum** should be used for cumulative quantities, such as carbohydrate intake or insulin doses, while the **mean** is appropriate for rates or continuous measurements, such as heart rate values. Some features may possess both a start and end timestamp. For example, a calorie burn entry might extend from 10:03 to 10:21, while the aggregation interval in the tabular format is set to 5 minutes. For such features, we recommend calculating the proportion of the value that falls within each time window and distributing the data according to the overlap with each interval.

Resampling should always employ right-aligned labeling, assigning measurements to the subsequent time point. This approach facilitates retrospective analysis at the prediction moment without introducing data leakage into future time steps.

**Fig. 2:**
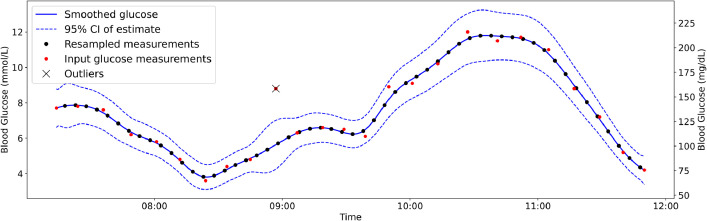
Illustration of input glucose measurements, including noise and irregular sampling, processed through a Kalman filter. Resampled values are extracted at 5-minute intervals. This figure is based on a Python-version of the software described in [13].

The CGM feature is critical, as it represents the target variable being predicted. Data sharers should retain the original CGM values in the dataset. However, an additional smoothed version of the CGM data could be included as a supplementary feature, along with metrics like the standard deviation of the probability that missing values were accurately imputed. We recommend using a Kalman filter for smoothing, as it provides accurate state estimates at each time point and employs a physiology-based approach to mitigate noise. Staal et al. proposed a Kalman smoothing filter specifically designed for blood glucose data, accompanied by open-source MATLAB code [13]. This filter has also been applied in a stacked LSTM model by Rabby et al., demonstrating improved prediction accuracy [14]. Fig. 2 illustrates the application of Kalman smoothing to refine the data, remove outliers, and resample it into 5-minute intervals. It is important to note that the Kalman smoothing filter is best suited for offline use. For real-time denoising of CGM measurements, online filtering methods, such as the approach suggested by Facchinetti et al. [15], are more appropriate.

From a data-sharing perspective, it is important to distinguish between truly missing values and the absence of events for event-based features, such as meal data. This distinction can be achieved by representing missing data with NaN (“Not a Number”) values and the absence of events with 0.0. Another important consideration is handling situations where actions, such as insulin delivery, are partially completed—e.g., when 4 U of insulin were intended, but delivery was canceled after 2.5 U. Data sharers should clearly document how such events are recorded and logged. It could be useful to include these events so that they can be analyzed specifically instead of only including the delivered amount. Researchers must pay attention to this documentation to ensure that they are correctly interpreting such events in the dataset for accurate analysis.

#### 1.4.8 Time zone handling

**Challenge:** Users moving across time zones complicates resampling data into a uniform time grid. This can lead to artificial gaps or errors in the data aggregation.

**Suggested solution:** To ensure uniformity, we recommend standardizing the datetime index to UTC+00:00. Including the local time zone as an additional field preserves context based on local time, allowing for the creation of derived time-based features, such as the hour of the day.

#### 1.4.9 Data validation

**Challenge:** When processing large datasets, it is easy to miss subjects with improperly formatted or faulty data values.

**Suggested solution:** After processing, systematically examine each feature and calculate the count of samples that fall outside a defined plausible range. For example, CGM values should not be negative, and a reasonable range might be between 10 and 720 mg/dL. Investigate any samples outside this range to determine if the deviation results from a processing error or represents a valid, though extreme, data point. Based on this evaluation, you may choose to adjust the processing, retain the sample, or remove it if it is conclusively erroneous. However, exercise caution, as legitimate outliers may contain valuable information and should not be discarded without thorough consideration.

## 2 Illustration of the Suggested Processing Approach

To illustrate our proposed processing approach, we have accessed three existing datasets: OhioT1DM [16] (including both the 2018 and 2020 versions), Tidepool Data [17], and T1DEXI [18]. Note that T1DEXI is divided into T1DEXI and T1DEXIP, with the latter representing pediatric subjects. The code for processing the datasets, as well as reproducing our results, is available at https://github.com/miriamkw/BGP_DatasetMerge. The code is written in Python, and we refer to the documentation in the repository for guidance on how to use it.

### 2.1 Resulting datasets overview

Table 1 provides an overview of the number of subjects and time spans of data for the datasets. Note that the counted samples are based on subjects using insulin pump therapy; the T1Dexi dataset includes more data when accounting for those using multiple daily injections. In addition, we have removed the subjects with no CGM values present. The sample count is based on the available CGM values, while a more detailed breakdown of the available features is provided in the next section. When we refer to “years of data,” we have calculated this based on the total number of CGM samples, given a sampling frequency of five minutes.

**Table 1:** An overview over the number of subjects and study length for the datasets.

Dataset	Number of unique subjects	Average time span for each subject (std)	Total samples
OhioT1DM	12	1.8 months (±0.1)	1.8 years
Tidepool	300	11.6 months (±7.6)	286.5 years
T1DEXI	409	0.9 months (±0.1)	30.6 years
T1DEXIP	211	0.3 months (±0.1)	5.9 years

**Total**	**932**	**4.6 months (±5.6)**	**324.8 years**

### 2.2 Features

Table 2 describes how each feature in the dataset was processed, aligning with our recommendations in the previous section.

**Table 2:** Details about how the features were processed with respect to our suggested guidelines.

Feature	Unit or scale	Processing
CGM	mg/dL	Ensuring consistent units
Carbs	Grams of carbohydrates	Cumulative sum within time intervals, using right labelling
Meal grams	Total grams of the meal	Cumulative sum within time intervals, using right labelling
Meal label	String description of the meal	Concatenation of strings within time intervals, using right labelling
Bolus	Insulin Units (IU)	Cumulative sum within time intervals, using right labelling
Basal	IU/h	Proportionally distributing the delivered insulin within the sampled time window
Galvanic skin response	Microsiemens	Aggregation using mean value within time interval, using right labelling
Skin temperature	Fahrenheit	Aggregation using mean value within time interval, using right labelling
Workout label	String	Concatenation of strings within time intervals, using right labelling
Workout intensity	Scale from 1 to 10	Mapping into the same scale, varying from strings, 0-2 and 1-10.
Steps	Count	Cumulative sum within time intervals, using right labelling
Acceleration	m/s^2^	Aggregation using mean value within time interval, using right labelling
Air temperature	Fahrenheit	Aggregation using mean value within time interval, using right labelling
Heartrate	Beats per minute	Aggregation using mean value within time interval, using right labelling
Calories burned	Kilocalories	Cumulative sum within time intervals, using right labelling

**Fig. 3:**
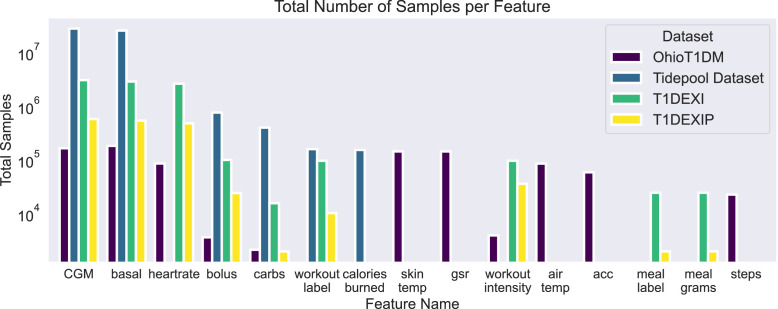
The total number of samples per feature for each dataset. The y-axis is displayed on a logarithmic scale to enhance the visibility of less frequent features. ‘Gsr’ denotes galvanic skin response, while ‘acc’ denotes acceleration.

Fig. 3 visualizes the number of samples for each feature in each dataset. The y-axis is on a logarithmic scale to improve visibility for less frequent features. As shown, CGM, basal rates, and bolus doses are present across all datasets. The Tidepool dataset generally contributes a large number of samples, while the Ohio T1DM dataset exhibits a more varied feature space, including features such as skin temperature, galvanic skin response, and step count. Notably, each dataset contains at least one feature from each category: blood glucose measurements, meals (e.g., carbohydrates, meal label, or meal grams), insulin (e.g., basal and bolus doses), and exercise (e.g., heart rate, workout label, or calories burned). As a consolidated dataset, this combination provides valuable data with potential for analysis and algorithm development.

### 2.3 Feature sparsity

**Fig. 4:**
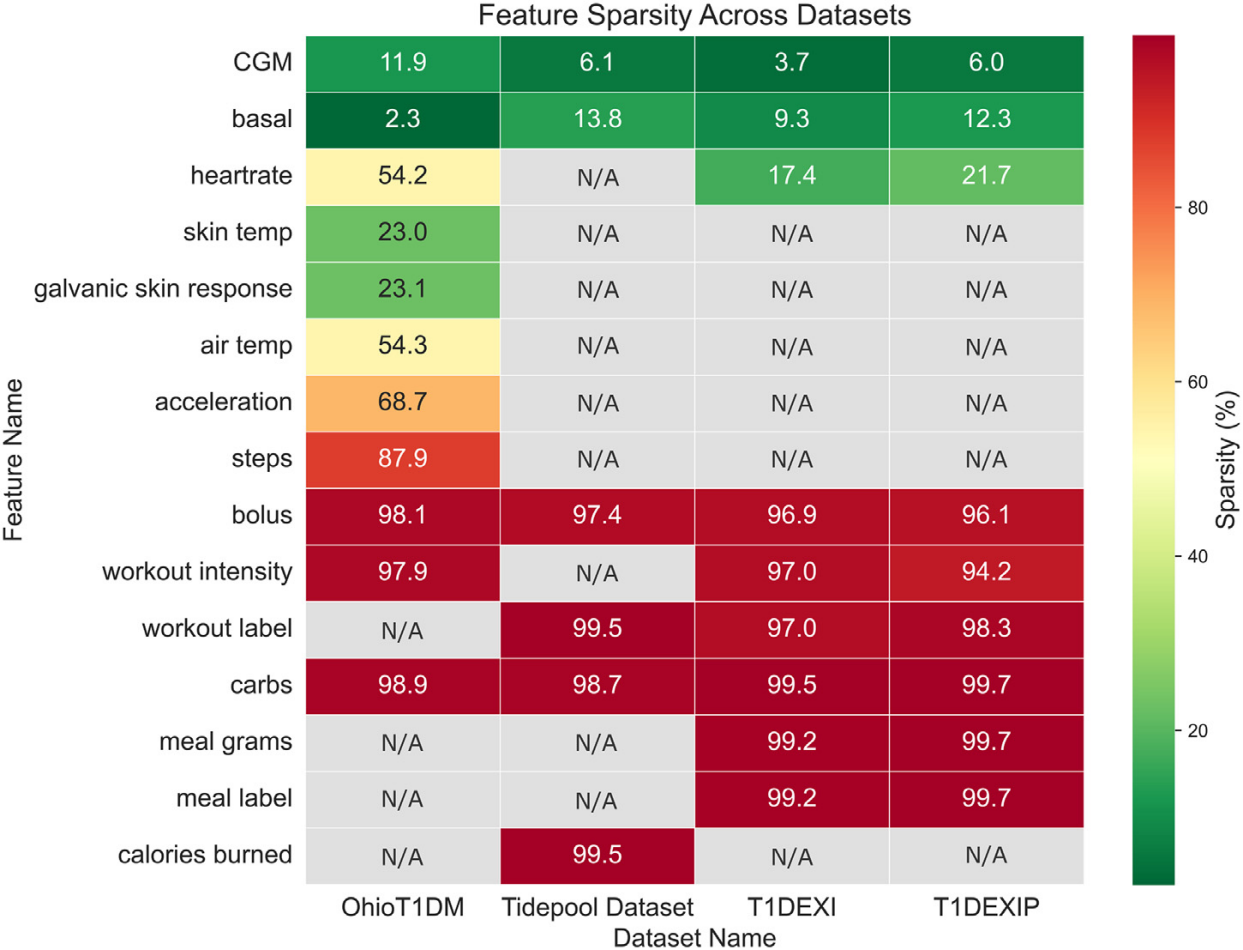
The feature sparsity across each dataset. The value within each cell represents the percentage of missing values for a given feature in a dataset. Grey cells indicate that the feature is unavailable (N/A) in the dataset.

In Fig. 4, we present the feature sparsity across the different datasets. Sparsity is defined as the percentage of missing values calculated on a consistent time grid, bounded by the first and last available CGM reading. The figure also highlights the availability of specific features within each dataset, with grey cells indicating the absence of a feature. The features *basal* and *CGM* exhibit consistently low sparsity across all datasets. Among the remaining features, *heart rate* is the most consistently available. Event-based features, such as *carbohydrates* or *workout intensity*, show high sparsity due to their occurrence at irregular intervals rather than every five minutes. Lastly, other biometric variables are not consistently present across the datasets.

## 3 Discussion

The integration of diverse diabetes management datasets, each with unique characteristics and varying sampling rates, presents several challenges. Research efforts often rely on single datasets for data analysis or algorithm development, limiting the generalizability of findings. Furthermore, inconsistencies in data processing make it difficult to compare results across studies. This paper proposes initial guidelines and recommendations for harmonizing multi-source data and emphasizes the need for a detailed consensus within the field to improve data-sharing practices.

Data quality poses multiple technical challenges in these datasets. First, sensor measurements are often prone to inaccuracies and noise. For example, CGM measurements are derived from interstitial fluid, which introduces a delay relative to blood glucose levels. Additionally, dietary information is frequently obtained through patient self-reporting, inherently prone to estimation errors, inaccurate timing, and occasional omissions. Basal insulin rates introduce further complexity; while typically recorded in units per hour (U/h), these are not delivered continuously but in intervals, such as every 30 minutes, with exact patterns sometimes unknown. Device variability is another factor, as the same feature, such as heart rate, may yield different values depending on the monitoring device used. Our recommendations address some of these challenges through approaches like Kalman smoothing and data aggregation. We also suggest that model developers apply normalization techniques, though we did not include normalization in preprocessing, as it is model-dependent. Nevertheless, despite these recommendations, data quality remains an inherent limitation.

We observed that certain subjects contributed substantially more data than others (Table 1). While subjects with extensive longitudinal data offer valuable opportunities for investigating seasonal patterns, long-term changes in blood glucose, and the potential for online learning techniques, this imbalance may also introduce bias toward these individuals in the analysis.

Depending on the dataset characteristics, there may be a need for flexibility in the selection of time intervals within the uniform time grid. In our analysis, we employed 5 minute intervals; however, this could be easily adjusted to suit the nature of the data or the study's specific requirements. For example, a finer time grid may be preferable when key features are sampled at higher rates, as it allows for a more granular representation of the data.

Imputation and other preprocessing techniques should be left to the user, as different use cases often require tailored approaches. However, when features exhibit lower sampling frequencies, users may need to employ imputation to address missing samples. It is essential to emphasize that in test data, two-way imputation should be avoided to prevent data leakage. Instead, missing samples in test datasets should either be excluded or handled using methods such as forward filling.

When datasets contain non-overlapping features, they can still be combined to enhance analysis by deriving new features or transforming existing ones. For instance, if one dataset includes the number of calories burned and another provides workout labels (e.g., “running” or “cycling”), these could be combined by estimating calorie expenditure for each unique workout label, or by assigning workout labels to calorie entries. Another potential to use various features is using feature-to-text transformation if the prediction model is based on a language model. However, combining datasets with non-overlapping features poses a challenge for machine learning model training, as models generally require a consistent set of input features. Future work could focus on improving the compatibility of datasets containing non-overlapping features.

A key limitation of this study is the lack of validation for our proposed harmonization guidelines, which are based on existing dataset formats. While these guidelines reflect the current landscape, they may not fully accommodate future developments. As datasets evolve with new innovations and features, our recommendations might become outdated, raising concerns about their long-term applicability.

## 4 Conclusion

This study provides initial guidance for preparing diabetes management datasets aimed at both dataset providers and researchers. We outline challenges such as integrating data from diverse sources, addressing inconsistencies in sampling frequencies, units, and scales, and managing data quality issues. To support these efforts, we propose a set of guidelines and demonstrate their application by processing and analyzing three datasets, with open access to the accompanying code for reproducibility and further exploration. However, one key limitation of this study is the lack of validation of our suggested guidelines, which raises concerns about their future applicability. Additionally, a challenge we encountered was the variation in features across datasets. Future work could address this limitation by aligning features to a consistent type and scale through feature derivation or data transformation. We encourage the research community to refine and expand upon our harmonization guidelines to establish detailed and standardized data-sharing practices. Such efforts can promote consistency, reduce the time and effort required for data preparation, and enable improved data integration, ultimately advancing the development of more effective diabetes management systems.

## Ethics Statement

This study utilized secondary data sources that contain human health-related data, which were collected by other researchers. We obtained access to these datasets through Data Use Agreements, ensuring compliance with ethical guidelines and confidentiality standards. The study did not involve direct interaction with human subjects, and therefore, no additional ethical approval was required. The authors confirm that they have read and follow the ethical requirements for publication in Data in Brief.

## Credit Author Statement

**Miriam K. Wolff:** Conceptualization, Methodology, Software, Writing – Original Draft. **Sam Royston:** Conceptualization, Investigation, Writing – Review & Editing. **Anders L. Fougner:** Writing – Review & Editing, Supervision. **Hans Georg Schaathun:** Writing – Review & Editing, Supervision. **Martin Steinert:** Supervision. **Rune Volden:** Supervision.

## Declaration of Generative AI and AI-assisted Technologies in the Writing Process

This article reflects the author's independent work. AI assistance has been employed (through ChatGPT) exclusively to the following ends: (i) to reformulate selected English sentences that included jargon or lacked clarity, (ii) as a thesaurus. An example prompt is “Please suggest an alternative formulation to the following sentences to improve readability, given that the context is academic writing: … ”. In all cases, both inputs and outputs from the tool have been meticulously refined by the authors before being included in the manuscript. The authors assert their moral rights as the creators of this piece, as well as their responsibility for it.

## References

[1] K. Saiti, M. Macas, K. Stechova, P. Pithova, and L. Lhotska, A review of model prediction in diabetes and of designing glucose regulators based on model predictive control for the artificial pancreas. 2017, p. 81. doi: 10.1007/978-3-319-64265-9_6.

[2] Cappon G., Prendin F., Facchinetti A., Sparacino G., Favero S.D. (Nov. 2023). Individualized models for glucose prediction in type 1 diabetes: comparing black-box approaches to a physiological white-box one. IEEE Trans. Biomed. Eng..

[3] “Public study websites.” Accessed: Oct. 31, 2024. [Online]. Available: https://public.jaeb.org/datasets/diabetes

[4] M. Martin et al., “irinagain/Awesome-CGM: Updated release with simulators and enhanced processing.” Accessed: Oct. 31, 2024. [Online]. Available: https://zenodo.org/records/4723654

[5] Cinar A., Turksoy K., Cinar A., Turksoy K. (2018). Advances in Artificial Pancreas Systems: Adaptive and Multivariable Predictive Control.

[6] Bailey R.L. (Mar. 2021). Overview of dietary assessment methods for measuring intakes of foods, beverages, and dietary supplements in research studies. Curr. Opin. Biotechnol..

[7] Hettiarachchi C., Daskalaki E., Desborough J., Nolan C.J., O'Neal D., Suominen H. (Feb. 2022). Integrating multiple inputs into an artificial pancreas system: narrative literature review. JMIR Diabetes.

[8] Saganowski S. (Dec. 2020). MobiQuitous 2020 - 17th EAI International Conference on Mobile and Ubiquitous Systems: Computing, Networking and Services.

[9] Cappon G., Vettoretti M., Sparacino G., Favero S.D., Facchinetti A. (Nov. 2023). ReplayBG: a digital twin-based methodology to identify a personalized model from type 1 diabetes data and simulate glucose concentrations to assess alternative therapies. IEEE Trans. Biomed. Eng..

[10] Contreras I., Oviedo S., Vettoretti M., Visentin R., Vehí J. (Nov. 2017). Personalized blood glucose prediction: a hybrid approach using grammatical evolution and physiological models. PLoS. One.

[11] Sun X. (2020). KDH@ECAI.

[12] Jacobs P.G. (Nov. 2023). Artificial intelligence and machine learning for improving glycemic control in diabetes: best practices, pitfalls and opportunities. IEEe Rev. Biomed. Eng..

[13] Staal O.M., Salid S., Fougner A., Stavdahl O. (Jan. 2019). Kalman smoothing for objective and automatic preprocessing of glucose data. IEEe J. Biomed. Health Inform..

[14] Rabby M.F., Tu Y., Hossen M.I., Lee I., Maida A.S., Hei X. (Mar. 2021). Stacked LSTM based deep recurrent neural network with Kalman smoothing for blood glucose prediction. BMC. Med. Inform. Decis. Mak..

[15] Facchinetti A., Sparacino G., Cobelli C. (May 2010). Enhanced accuracy of continuous glucose monitoring by online extended kalman filtering. Diabetes. Technol. Ther..

[16] Marling C., Bunescu R. (Sep. 2020). The OhioT1DM dataset for blood glucose level prediction: update 2020. CEUR. Workshop. Proc..

[17] Bartolome A., Prioleau T. (Aug. 2022). A computational framework for discovering digital biomarkers of glycemic control. NPJ. Digit. Med..

[18] Riddell M.C. (Feb. 2023). Examining the acute glycemic effects of different types of structured exercise sessions in Type 1 diabetes in a real-world setting: the type 1 diabetes and exercise initiative (T1DEXI). Diabetes Care.

